# Management of thrombocytopenia in the ICU (pregnancy excluded)

**DOI:** 10.1186/2110-5820-2-42

**Published:** 2012-08-28

**Authors:** Thierry Van der Linden, Bertrand Souweine, Laurent Dupic, Lilia Soufir, Pascal Meyer

**Affiliations:** 1 Service de Réanimation Polyvalente, groupe hospitalier Institut Catholique de Lille/Faculté Libre de Médecine/Université Lille Nord de France, F-59462 Lomme lez Lille, France; 2 Service de Réanimation Polyvalente, CHU Gabriel Montpied, 58 rue Montalembert, F-63003 Clermont-Ferrand, France; 3 Service de Réanimation Médico-Chirurgicale, CHU Necker Enfants Malades, 149, Rue de Sèvres, 75743 Paris cedex 15, France; 4 Service de Réanimation Chirurgicale, Groupe Hospitalier Paris Saint Joseph, 85 rue Raymond Losserand, 75014 Paris, France; 5 Service de Réanimation Polyvalente, CH Sud Francilien Site de Corbeil, 59, Boulevard Henri Dunant, 91106 Corbeil-Essonnes cedex, France

**Keywords:** Thrombocytopenia, Critical care, Adults, Expert recommendations

## Abstract

Thrombocytopenia is a very frequent disorder in the intensive care unit. Many etiologies should be searched, and therapeutic approaches differ according to these different causes. However, no guideline exists regarding optimum practices for these situations in critically ill patients. We present recommendations for the management of thrombocytopenia in intensive care unit, excluding pregnancy, developed by an expert group of the French-Language Society of Intensive Care (Société de Réanimation de Langue Française (SRLF), the French Language Group of Paediatric Intensive Care and Emergencies (GFRUP) and of the Haemostasis and Thrombosis Study Group (GEHT) of the French Society of Haematology (SFH). The recommendations cover six fields of application: definition, epidemiology, and prognosis; diagnostic approach; therapeutic aspects; thrombocytopenia and sepsis; iatrogenic thrombocytopenia, with a special focus on heparin-induced thrombocytopenia; and thrombotic microangiopathy.

## Review

### Introduction and presentation of the expert recommendations of the French-language Intensive Care Society (Société de Réanimation de Langue Française (SRLF))

Thrombocytopenia is the hemostatic disorder that is most frequently encountered in intensive care and is seen in 41–66% of patients (close to 50% at admission). In view of the mortality associated with thrombocytopenia, the numerous associated pathological conditions seen in intensive care, and the lack of recommended treatment strategy, the SRLF has drawn up these recommendations.

An SRLF expert group drew up recommendations, giving reasoned arguments for each of six fields of application defined by the organizing committee, including pediatric specificities. Each expert (or expert subgroup) then presented and justified the content and form of the proposed recommendations, changes to which could be made during the ensuing discussion. The revised recommendations were then put to the vote. The purpose was not to impose a single expert point of view for all the recommendations but to delineate clearly the points of agreement that underpin the recommendations, as well as points of disagreement or indecision that may form the basis of future work.

Recommendations were scored according to an approach based on the RAND/UCLA Appropriateness Method. After the first round of voting for each recommendation (score 1 for complete disagreement or lack of any proof to 9 for complete agreement or conclusive evidence), the highest and lowest scores were eliminated, and the score was taken as the median of the remaining scores. The upper and lower bounds were then considered as “strong agreement” if they lay within the range 7–9 and “strong disagreement” in the range 1–3. Such scores were considered definitive. Recommendations whose scores were not within these two ranges were voted on again, following the same principle. After this second round of voting, the scores were taken as follows:

– Median and bounds within range [7–9] = “strong agreement”

– Median and bounds within range [1–3] = “strong disagreement”

– Median within range [7–9] with lower bound < 7 = “weak agreement”

– Median within range [1–3] with upper bound > 3 = “weak disagreement”

– Median within range [4–6] = “uncertainty”

The methodology used was inspired by the GRADE system (Grading of Recommendations Assessment, Development and Evaluation) for rating clinical guidelines (http://www.gradeworkinggroup.org/links.htm), the originality of which stems primarily from three assertions. First, characterization of the type of study alone (e.g., randomized or not) is insufficient to indicate the level of proof of a study. Second, the risk/benefit ratio is taken into account. Third, the exact wording of guidelines carries clear implications for users: “should be done/recommended…” (strong agreement) or “should not be done/not recommended” (strong disagreement), “should probably be done/is possible” (weak agreement), or “should probably not be done/is possible not to do” (weak disagreement).

#### Field 1: Definition – epidemiology – prognosis

1. Thrombocytopenia is defined by a platelet count (PC) < 150 × 10^9^/L *(strong agreement).*

2. In intensive care, pseudothrombocytopenia should be discounted using *in vitro* agglutination of platelets, by preparing a blood film, or, if necessary, using a blood sample collected in a citrate tube *(strong agreement).*

3. An etiological investigation is required to determine the causes and underlying mechanisms of a PC < 100 × 10^9^/L or a > 30% decrease in PC. It also can be done for a drop in PC, independently of these cutoffs, depending on the pathological context *(strong agreement).*

4. A more than 30% drop in PC should probably be considered to be associated with a poor prognosis *(weak agreement).*

5. Changes in PC over time (duration, speed of decrease or rise, etc.) should be considered to be associated with the prognosis *(strong agreement).*

#### Field 2: Diagnostic approach

6. The underlying mechanisms and etiologies of thrombocytopenia in intensive care are often multiple, with peripheral causes predominating *(strong agreement).*

7. Sepsis is the main etiology of thrombocytopenia in intensive care and is usually associated with disseminated intravascular coagulation (DIC) *(strong agreement).*

8. Diagnosis of DIC should be based on both clinical and biochemical criteria. International Society on Thrombosis and Haemostasis (ISTH) and Japanese Association for Acute Medicine (JAAM) scores can be used to objectify DIC (scores in Additional file 1*and*2*) (strong agreement).*

9. The clinical context should suggest the etiology of thrombocytopenia in most cases in intensive care *(strong agreement).*

10. In thrombocytopenia in intensive care, bone marrow biopsy should not be performed routinely but can be considered when there is no obvious etiology or when other cell lineages are affected *(strong agreement).*

11. Screening for antiplatelet antibodies is unjustified in the initial diagnostic approach to thrombocytopenia *(strong agreement).*

12. It is important to know which diagnoses of thrombocytopenia justify special investigations and prompt initiation of specific treatments (notably thrombotic microangiopathy [TMA], macrophage activation syndrome, catastrophic antiphospholipid syndrome) *(strong agreement).*

#### Field 3: Therapeutic aspects

13. In intensive care, bleeding severity can be graded with a 5-point scale used in the literature *[0: no hemorrhage; 1: slight hemorrhage; 2: patent blood loss not requiring red cell transfusion; 3: blood loss requiring red cell transfusion; 4: hemorrhage with considerable morbidity] (strong agreement).*

14. For a similar PC, central thrombocytopenia probably involves a greater risk of bleeding than peripheral thrombocytopenia. Bleeding complications occur above all with central thrombocytopenia < 20 × 10^9^/L *(weak agreement).*

15. The decision to treat thrombocytopenia should be based on the PC but also on the:

• Presence of active bleeding (type, potential severity),

• Mechanism of thrombocytopenia (central or peripheral),

 • Etiology,

• Risk of thrombosis,

• Risk of hemorrhage (platelet disorders, invasive procedures or surgery), and

• Associated treatments *(strong agreement).*

16. If platelet transfusion is necessary, pooled standard platelet units should be used, except for allo-immunized patients, those receiving treatment for blood disorders, and those with blood disorders, all of whom should be given aphaeresis platelet concentrate (single donor)*(strong agreement).*

17. The number of platelet units transfused should be prescribed depending on body weight: 1 platelet unit (0.5 to 0.7 × 10^11^ platelets) per 7 kg body weight in adults and per 5 kg in children *(strong agreement).*

18. ABO/Rh1 (D)-compatible platelets should be preferred for transfusion *(strong agreement).*

19. There is no need to calculate the percent platelet recovery *(strong agreement).*

20. Depending on the context and in the event of severe risk of hemorrhage, it is probably necessary to measure the PC in the hour following the end of platelet transfusion *(weak agreement).*

21. The risk of hemorrhage in thrombocytopenia should not be defined by the PC alone, but by a combination of PC < 50 × 10^9^/L, the clinical situation and factors that may influence primary or secondary hemostasis *(strong agreement).*

22. In intensive care, prophylactic platelet transfusion should not be routine when the PC > 20 × 10^9^/L in central or peripheral thrombocytopenia *(strong agreement).*

23. In intensive care, prophylactic platelet transfusion should probably be done in central thrombocytopenia when the PC < 20 × 10^9^/L *(weak agreement).*

24. In intensive care, in the case of severe hemorrhage, platelet transfusion is recommended when the PC ≤ 50 × 10^9^/L *(strong agreement).*

25. In intensive care, platelet transfusion is probably necessary when the PC ≤ 50 × 10^9^/L in the following situations:

– Severe sepsis, with risk of severe hemorrhage or use of anticoagulant,

– Invasive procedure,

– Pre- or postsurgical setting *(weak agreement).*

26. In intensive care, platelet transfusion is probably necessary when the PC ≤ 100 × 10^9^/L: after surgery of the central nervous system, liver, eye, and large blood vessels; in patients with polytrauma *(weak agreement).*;

27. In intensive care, prophylactic platelet transfusion is not recommended in the following situations:posttransfusion purpura, thrombotic thrombocytopenic purpura (TTP), catastrophic antiphospholipid syndrome, hemolytic-uremic syndrome (HUS), heparin-induced thrombocytopenia (HIT) *(strong agreement).*

28. In intensive care, prophylactic platelet transfusion is probably not recommended in cases of DIC *(weak agreement).*

29. In intensive care, the risk of hemorrhage (severity of thrombocytopenia, associated platelet disorders) and the risk of thromboembolic events should be assessed before deciding to administer antiplatelet drugs, curative anticoagulant treatment, or preventive anticoagulant treatment to thrombocytopenic patients *(strong agreement).*

30. Antithrombotic prophylaxis using unfractionated heparin (UFH) or low-molecular-weight heparin (LMWH) should probably be prescribed routinely in all adult patients admitted to intensive care, except when the PC < 30 × 10^9^/L or there is a major risk of hemorrhage *(strong agreement).*

31. Any interruption of antiplatelet drugs in an intensive care patient should be as short as possible, especially if the patient has a drug-eluting stent *(strong agreement).*

32. When a patient admitted to intensive care is already taking antiplatelet drugs, these should probably be withdrawn if there is a risk of hemorrhage (PC < 50 × 10^9^/L, other risk factors, etc.) *(weak agreement).*

#### Field 4: Thrombocytopenia and sepsis

34. Use of immunoglobulins in thrombocytopenic patients with sepsis is not recommended *(strong agreement).*

35. Thrombocytopenia is very frequent in infectious purpura fulminans, because DIC is more severe that in other septic shock etiologies in children *(strong agreement).*

36. Apart from immediate parenteral administration of a first dose of antibiotic as soon as infectious purpura fulminans is suspected, there is no recommended treatment specific to infectious purpura fulminans compared with other septic shock etiologies in children*(strong agreement).*

37. For infectious thrombocytopenia, routine administration of treatments specific to hemostasis (protein C, antithrombin III, anticoagulants, fibrinolytics) is not recommended because of the high risk of hemorrhage. It can be considered in the most severe cases, always following individualized reassessment of the risk/benefit ratio *(strong agreement).*

#### Field 5: Iatrogenic thrombocytopenia

38. Etiological diagnosis of drug-induced thrombocytopenia in intensive care should be guided by its origin (peripheral versus central) and mechanism (immune versus nonimmune) *(strong agreement).*

39. HIT should probably be suspected for any thrombocytopenia in intensive care (*weak agreement).*

40. Drug-induced thrombocytopenia should be treated by withdrawal of the drug concerned, which retrospectively confirms the diagnosis *(strong agreement).*

41. In very severe forms (PC < 5 × 10^9^/L or with life-threatening bleeding) of drug-induced thrombocytopenia with an immune mechanism, intravenous immunoglobulins or even plasmapheresis is possible *(weak agreement).*

42. Thrombocytopenia after chemotherapy most often is linked to myelosuppression, its severity and duration depends on the type and dose of cytostatic drugs used and on the initial PC *(strong agreement).*

43. In central thrombocytopenia after chemotherapy, platelet transfusion is recommended for a PC < 20 × 10^9^/L with signs of bleeding and should be routine for a PC < 10 × 10^9^/L *(strong agreement).*

44. Severe thrombocytopenia in the multiparous woman or the patient who has received multiple transfusions is indicative of posttransfusion purpura, which can be treated with intravenous immunoglobulins *(strong agreement).*

45. For patients at high risk of hemorrhage without liver failure, citrate should be the anticoagulant of choice for intermittent and continuous renal dialysis *(strong agreement).*

46. Extracorporeal membrane oxygenation (ECMO) or extracorporeal life support (ECLS) in intensive care is always accompanied by a decrease in PC and requires anticoagulation, generally with low doses of heparin (5–20,000 IU/day) for the first few days, with dosage adjustment according to hemostasis tests *(strong agreement).*

#### Heparin-induced thrombocytopenia

47. Whichever heparin is used (UFH or LMWH), baseline PC should be measured on initiation or during the first 24 hours of treatment *(strong agreement).*

48. PC should probably be measured twice in any patient exposed to UFH during the past 3 months, first upon initiation of heparin therapy (UFH or LMWH) and then the next day *(weak agreement).*

49. In intensive care, PC should be measured twice a week during the first 3 weeks of heparin therapy (UFH or LMWH)*(strong agreement).*

50. Warkentin 4-T score should be used to assess the probability of HIT, even though it has not been specifically validated in intensive care (see Additional file 3) *(strong agreement).*

51. In patients with a Warkentin 4-T score < 2, a laboratory test of HIT should probably not be done *(weak agreement).*

52. Two types of diagnostic laboratory tests should probably be combined in any patient with suspected HIT: one functional and one immunoenzymatic (ELISA) to screen for antiheparin PF4 antibodies *(weak agreement).*

53. Heparin should be excluded if there is a history of HIT. A nonheparin anticoagulant is recommended *(strong agreement).*

54. If there is a history of HIT, danaparoid is the first-line anticoagulant in intensive care *(strong agreement).*

55. In a patient with suspected HIT and a Warkentin 4-T score ≥ 2, withdrawal of heparin should probably be considered *(weak agreement).*

56. In a patient with suspected HIT and a Warkentin 4-T score ≥ 4, heparin should be replaced immediately by an empirical nonheparin treatment at curative dosage: danaparoid sodium, lepirudin, or even bivalirudin *(strong agreement).*

57. In intensive care, fondaparinux is probably not recommended as replacement therapy during the acute phase of HIT *(weak agreement).*

58. In intensive care, the introduction of vitamin K antagonists during the acute phase of HIT is contraindicated *(strong agreement).*

#### Field 6: Thrombotic microangiopathy

59. Thrombocytopenia associated with mechanical hemolytic anemia should suggest TMA, even in the absence of organ failure *(strong agreement).*

60. In any patient with suspected TMA, the blood samples (2 dry tubes and 2 EDTA tubes) for etiological investigation (ADAMTS13 activity, complement, autoimmunity, HIV) should be collected before any treatment, without delaying its initiation *(strong agreement).*

61. The first-line treatment of TMA should be based on emergency plasma exchange (PE) (rate 60 mL/kg). If PE is not initially possible, plasma should be infused at 20 mL/kg when possible *(strong agreement).*

62. In TMA, PE should be continued daily for at least 5 to 7 days until the PC normalizes and is stable for at least 48 hours. The rate of PE is lowered progressively, on a case-by-case basis *(strong agreement).*

63. In adults and children with TMA, platelet transfusion is strictly contraindicated unless there is life-threatening bleeding *(strong agreement).*

64. In adults with TMA, an invasive procedure is not an indication for routine prophylactic platelet transfusion because of potentially lethal adverse effects *(strong agreement).*

65. In children with TTP, an invasive procedure is probably not an indication for routine prophylactic platelet transfusion because of potentially lethal adverse effects *(weak agreement).*

66. In children with HUS, routine prophylactic platelet transfusion should be restricted to invasive procedures that have a high risk of hemorrhage *(strong agreement).*

67. For the specific treatment of TMA, infusion of heparin, fibrinolytics, prostacyclin, or vitamin E is useless, even dangerous. There is no evidence for the efficacy of antiplatelet drugs *(strong agreement).*

68. In TTP, patients should be routinely monitored and tested for myocardial ischemia *(strong agreement).*

69. In TTP, methylprednisolone can be used if not contraindicated, in combination with PE *(strong agreement).*

70. In congenital TTP, specific treatment should be based on administration of plasma (10 mL/kg every 2 to 4 weeks) *(strong agreement).*

71. In HUS, stools or rectal swabs should be tested for Shiga toxin-producing *E. coli* (STEC) using culture on MacConkey agar and polymerase chain reaction to test for Shiga toxin *(strong agreement).*

72. In HUS, if no STEC is found in the stools, the serum should probably be tested for anti-LPS IgM of the most frequent serogroups of STEC *(weak agreement).*

73. Children with HUS should be transferred to a specialized department (nephrology or intensive care) for discussion of prompt initiation of hemodialysis *(strong agreement).*

74. In adults and children with HUS caused by STEC, PE has not proven effective and should probably not be recommended *(weak agreement).*

75. PE should probably be used in HUS caused by STEC with central neurological involvement *(weak agreement).*

76. In HUS caused by *Streptococcus pneumoniae*, administration of plasma is probably contraindicated and only washed packed red blood cells or platelets should be used when the agglutination test is positive*(weak agreement).*

77. First-line treatment of atypical HUS should be emergency PE (rate 60 mL/kg). If PE is not initially possible, plasma should be infused at 10–20 mL/kg when possible *(strong agreement).*

78. If the response to first-line treatment is suboptimal, immunomodulators should be considered (rituximab in acquired TTP, eculizumab in atypical HUS), in collaboration with a reference center. Suboptimal response is defined as follows: 

– Atypical HUS: failure to correct thrombocytopenia and/or to improve renal function at day 5 of standard treatment 

– Acquired TTP: PC not doubled by day 5 of standard treatment or diseaseprogression (increased thrombocytopenia or onset of organ damage) duringintensive first-line treatment or on reducing the frequency of PE *(strong agreement).*

79. In the absence of treatment, the prognosis of atypical HUS is guarded (death in 10% of cases and progression to terminal renal failure in 50% of cases), particularly when there is factor H mutation (death or terminal renal failure immediately or within 1 year in 70% of cases) *(strong agreement).*

#### Uncertainty

#### The experts’ scores for the following statements lay within a range of uncertainty

PC < 150 × 10^9^/L is associated with an unfavorable prognosis.

In intensive care, prophylactic platelet transfusion should be performed in cases of peripheral thrombocytopenia < 10 × 10^9^/L.

## Abbreviations

DIC: disseminated intravascular coagulation; HIT: heparin-induced thrombocytopenia; HUS: hemolytic-uremic syndrome; LMWH: low-molecular-weight heparin; PC: platelet count; PE: plasma exchange; STEC: Shiga toxin-producing *E. coli *; TMA: thrombotic microangiopathy; TTP: thrombotic thrombocytopenic purpura; UFH: unfractionated heparin.

## Competing interests

The authors and the experts declare that they have no competing interests.

## Authors’ contributions

TV, BS, LD, LS, and PM contributed to the conception and design of the study, the draft, and critical revision of the manuscript, and all authors approved the final version.

## Authors’ information

1. Organising Committee for the SRLF Recommendations and Assessments Board: M. Monchi, C. Bretonnière, T. Boulain, K. Chaoui, A. Cravoisy, D. Da Silva, M. Djibré, F. Fieux, D. Hurel, V. Lemiale, O. Lesieur, M. Lesny, P. Meyer, C. Milesi, B. Misset, D. Orlikowski, D. Osman, JP. Quenot, L. Soufir, T. Van der Linden, I. Verheyde (T. Van der Linden).

2. Expert Coordinator (B. Souweine).

3. Experts: N. Ajzemberg (Paris), P. Coppo (Paris), M. Darmon (Saint Etienne), S. Dauger (Paris), L. Drouet (Paris), I. Elalamy (Paris), B. François (Limoges), D. Gruson (Bordeaux), C. Loirat (Paris), B. Megarbane (Paris), F. Pene (Paris), B. Souweine (Clermont-Ferrand), F. Stephan (Marie Lannelongue), B. Tardy (Saint Etienne) (group of experts).

Expert recommendations formalized under the aegis of the French-language Intensive Care Society (Société de Réanimation de Langue Française; SRLF), with the participation of the French Language Group of Paediatric Intensive Care and Emergencies (GFRUP) and of the Haemostasis and Thrombosis Study Group (GEHT) of the French Society of Haematology (SFH).

## Supplementary Material

Additional file 1 ** Diagnostic algorithm for overt DIC according to the International Society on Thrombosis and Haemostasis (ISTH), from Taylor Jr FB et al *Thromb Haemost 2001. *** Click here for file

Additional file 2 ** Diagnostic score for DIC according to the Japanese Association for Acute Medicine (JAAM). from Gando S. *Crit Care Med 2006. *** Click here for file

Additional file 3 ** Clinical probability of HIT according to the Warkentin 4-T score. from Warkentin TE et al., *Hematology (Am Soc Hematol Educ Program) 2003. *** Click here for file

