# Supplementary material for: Management of thrombocytopenia in the ICU (pregnancy excluded)
Source: Ann Intensive Care. 2012 Aug 28;2:42. doi: 10.1186/2110-5820-2-42 (PMC3488545; doi:10.1186/2110-5820-2-42)
Supplement: Additional file 1 — Diagnostic algorithm for overt DIC according to the International Society on Thrombosis and Haemostasis (ISTH), from Taylor Jr FB et al Thromb Haemost 2001. [file 2110-5820-2-42-S1.doc]

**Appendix 1**

Diagnostic algorithm for overt DIC according to the International Society on Thrombosis and Haemostasis (ISTH), from Taylor Jr FB et al. *Thromb Haemost 2001*.

1. Risk assessment: does the patient have an underlying disorder known to be associated with DIC ?
   - if so: do the test
   - if not, do not use this algorithm
2. Perform coagulation tests [platelet count, prothrombin time, fibrinogen, markers of fibrin degradation *(fibrin degradation products, D-dimers, soluble fibrin monomers)*]
3. Assess the test results:
   - Platelets (> 100 = 0; < 100 = 1; < 50 = 2)
   - Markers of fibrin degradation (no increase: 0; moderate increase: 2; strong increase: 3)
   - Prolongation of prothrombin time (< 3 seconds = 0; from 3 to 6 seconds = 1; > 6 seconds = 2)
   - Fibrinogen level (> 1 g/L = 0; < 1 g/L = 1)
4. Add the scores:
   - If total ≥ 5: compatible with overt DIC : calculate the score daily.
   - If total < 5: suggests but does not confirm overt DIC : repeat the test after 24-48 hours.
