# Supplementary material for: Management of thrombocytopenia in the ICU (pregnancy excluded)
Source: Ann Intensive Care. 2012 Aug 28;2:42. doi: 10.1186/2110-5820-2-42 (PMC3488545; doi:10.1186/2110-5820-2-42)
Supplement: Additional file 2 — Diagnostic score for DIC according to the Japanese Association for Acute Medicine (JAAM). from Gando S. Crit Care Med 2006. [file 2110-5820-2-42-S2.doc]

**Appendix 2**

Diagnostic score for DIC according to the Japanese Association for Acute Medicine (JAAM). from Gando S. *Crit Care Med 2006*.

Points

Systemic inflammatory response criteria

≥ 3 1

0 - 2 0

Platelet count (10^9^/L)

< 80 or decrease > 50% in 24 hours 3

≥ 80 and <120 or decrease > 30% in 24 hours 1

≥ 120 0

Fibrinogen (g/L)

< 3.5 1

≥ 3.5 0

Prothrombin time (patient/control ratio)

≥ 1.2 1

< 1.2 0

Fibrin degradation products (mg/L)

≥ 25 3

≥ 10 and < 25 1

< 10 0

**DIC if the score is *≥* 5**
