# Supplementary material for: Management of thrombocytopenia in the ICU (pregnancy excluded)
Source: Ann Intensive Care. 2012 Aug 28;2:42. doi: 10.1186/2110-5820-2-42 (PMC3488545; doi:10.1186/2110-5820-2-42)
Supplement: Additional file 3 — Clinical probability of HIT according to the Warkentin 4-T score. from Warkentin TE et al., Hematology (Am Soc Hematol Educ Program) 2003. [file 2110-5820-2-42-S3.doc]

**Appendix 3:**

Clinical probability of HIT according to the Warkentin 4-T score.

from Warkentin TE et al, *Hematology (Am Soc Hematol Educ Program) 2003*.

| **Thrombocytopenia** | > 50% or nadir ≥ 20 x 10^9^/L | **2** |
| --- | --- | --- |
|  | ↓ 30 – 50% or nadir 10 – 19 x 10^9^/L | **1** |
|  | ↓ < 30% or nadir <10 x 10^9^/L | **0** |
| **Timing of thrombocytopenia** | Day 5-Day 10 or ≤ Day 1 + exposure ≤ 30 days | **2** |
|  | > Day 10 or ≤ Day 1 + exposure 31−100 days  or timing uncertain (complete blood count missing) but compatible | **1** |
|  | < Day 4 without exposure < 100 days | **0** |
| **Thrombosis or clinical sign** | New thrombosis documented, skin necrosis  or acute systemic reaction after IV bolus of UFH | **2** |
|  | Extension or recurrence of thrombosis or suspected undocumented thrombosis  Or erythematous plaques at injection point | **1** |
|  | None | **0** |
| **AnoTher cause of thrombocytopenia** | None obvious | **2** |
|  | Possible | **1** |
|  | Definite | **0** |

**Probability of HIT as a function of total score before the tests: 6-8: high**

**4-5: intermediate**

**0-3: low**
